# Supplementary material for: Effectiveness of Statins for Primary Prevention of Cardiovascular Disease in Low- and Medium-Risk Males: A Causal Inference Approach with Observational Data
Source: J Pers Med. 2022 Apr 20;12(5):658. doi: 10.3390/jpm12050658 (PMC9146525; doi:10.3390/jpm12050658)
Supplement: Supplementary file 1 [file jpm-12-00658-s001.zip › jpm-1609945-supplementary.pdf]

Supplementary Materials

Table S1. Number of subject-trials according the type of statin prescribed.

	**Number of subject-trials N = 473**
Low-moderate-intensity	
Simvastatin (C10AA01)	198
Lovastatin (C10AA02)	1
Pravastatin (C10AA03)	10
Fluvastatin (C10AA04)	5
High-intensity	
Atorvastatin (C10AA05)	89
Rosuvastatin (C10AA07)	126
Pitavastatin (C10AA08)	36
Combinations	
Simvastatin and ezetimibe (C10BA02)	5
Pravastatin and fenofibrate (C10BA03)	2
Atorvastatin and ezetimibe (C10BA05)	1

N, total number of treated subjects.
